# Cortical response of the ventral attention network to unattended angry facial expressions: an EEG source analysis study

**DOI:** 10.3389/fpsyg.2014.01498

**Published:** 2014-12-19

**Authors:** Alberto Inuggi, Federica Sassi, Alejandro Castillo, Guillermo Campoy, Letizia Leocani, José M. García Santos, Luis J. Fuentes

**Affiliations:** ^1^Basque Center on Cognition, Brain and LanguageSan Sebastián, Spain; ^2^Departamento de Psicología Básica y Metodología, University of MurciaMurcia, Spain; ^3^Institute of Experimental Neurology, L’Istituto di Ricovero e Cura a Carattere Scientifico San RaffaeleMilan, Italy; ^4^Servicio de Radiología, Hospital Morales MeseguerMurcia, Spain

**Keywords:** ventral attentional network, temporo–parietal junction, EEG source analysis, threatening facial expressions, attention modulation

## Abstract

**Introduction:** We used an affective prime task composed of emotional (happy, angry, and neutral) prime faces and target words with either positive or negative valence. By asking subjects to attend to either the faces’ emotional expression or to the glasses’ shape, we assessed whether angry facial expressions were processed when they were unattended and task-irrelevant.

**Methods:** We conducted a distributed source analysis on the corresponding event-related potentials focused on the early activity of face processing and attention networks’ related areas. We also evaluated the magnitude of the affective priming effect.

**Results:** We observed a reduction of occipitotemporal areas’ (BA37) activation to unattended compared to attended faces and a modulation of primary visual areas’ activity lateralization. The latter was more right lateralized for attended than for unattended faces, and emotional faces were more right lateralized than neutral ones only in the former condition. Affective priming disappeared when emotional expressions of prime faces were ignored. Moreover, an increased activation in the right temporo–parietal junction (TPJ), but not in the intraparietal sulcus, was observed only for unattended angry facial expressions at ∼170 ms after face presentation.

**Conclusion:** We suggest that attentional resources affect the early processing in visual and occipito-temporal areas, irrespective of the faces’ threatening content. The disappearance of the affective priming effect suggests that when subjects were asked to focus on glasses’ shape, attentional resources were not available to process the facial emotional expression, even though emotion-relevant and emotion-irrelevant features of the face were presented in the same position. On the other hand, unattended angry faces evoked a pre-attentive TPJ activity, which most likely represents a bottom–up trigger that signals their high behavioral relevance, although it is unrelated to task demands.

## INTRODUCTION

Emotional events play a crucial role in how humans interact with one another and how they can adapt to changing environments. To foster survival, it is essential that threatening stimuli that originate from other people or from the environment may be processed in a rapid and efficient manner. Many pieces of evidence show that threatening information can be processed automatically and independently of attention or attentional resources (Stenberg et al., 1995; Vuilleumier et al., 2001; for reviews, see Compton, 2003; Vuilleumier, 2005). Moreover, this information processing can occur even without conscious perception (for a recent review, see Tamietto and de Gelder, 2010).

One common stimulus used to demonstrate how threatening information can be prioritized and processed efficiently is the fearful facial expression. Several studies using different paradigms have shown that even though the emotional content of the stimulus is task-irrelevant, it captures attention and interferes with the relevant task (Okon-Singer et al., 2007; Hart et al., 2010), delays disengagement of attention (Georgiou et al., 2005), is detected more easily than a neutral stimulus (Hansen and Hansen, 1988; Anderson, 2005; Calvo et al., 2006) and is better detected as a T2 in the attention blink paradigm compared with a neutral T2 (Anderson, 2005). Further evidence for the automatic processing of emotional expressions is derived from studies that explicitly manipulated the focus of attention by asking subjects to either attend to or ignore facial stimuli [e.g., Vuilleumier et al., 2001; Anderson et al., 2003; Eimer et al., 2003; see Eimer and Holmes, 2007, for a review of event-related potential (ERP) studies]. For instance, Vuilleumier et al. (2001) presented two faces and two houses arranged parafoveally in the vertical or horizontal axis. Subjects had to compare the faces (faces-attended, houses-unattended) or to compare the houses (faces-unattended, houses-attended). Fearful faces were compared with neutral faces. The activation in the amygdala, the hallmark of emotional processing, was higher with fearful than with neutral faces. Notably, activation in the amygdala did not differ whether the participants paid attention to the faces or to the houses.

Recent studies, however, have challenged the idea that the processing of emotional information can occur without requiring a sufficient amount of attentional resources (Pessoa et al., 2002, 2005; Holmes et al., 2003; Ochsner and Gross, 2005; Okon-Singer et al., 2007; Silvert et al., 2007; Sassi et al., 2014). For instance, Pessoa et al. (2002; see also Pessoa et al., 2005) found emotion-related brain activity only when the subjects had to respond to the gender of the faces (easy task), but not when they had to discriminate the orientation of two peripheral bars (difficult task). Holmes et al. (2003) compared ERPs between fearful and neutral facial expressions when the subjects had to compare two faces (face attended) versus two houses (face unattended), with both faces and houses being simultaneously presented at different spatial locations. Differences between the two emotional expressions were observed only when faces were attended. In a recent behavioral study, Sassi et al. (2014) used an affective priming task in which a prime face showing either an emotional (positive or negative) or a neutral expression was followed by an emotionally laden target word (positive or negative). In the critical trials the target word could be preceded by a face prime that belonged to the same affective category of the target (congruent condition) or to a different affective category (incongruent condition). Affective priming was measured through congruency effects, that is, the difference in performance between the congruent condition and the incongruent condition. Sassi et al. (2014) observed affective priming when the subjects’ attention was allocated to the emotional information (emotion task), and also, albeit of a smaller size, when the emotion expression was made task-irrelevant by asking subjects to determine whether the face wore glasses (the glasses task). However, when the subjects were asked to determine whether the glasses were rounded or squared (the shape task), the affective priming effect vanished. This finding was probably a consequence of the fact that the shape task (difficult task) required more attentional monitoring than the glasses task (easy task), and therefore there were not sufficient attentional resources as to process the emotional expression of the face prime (see also Okon-Singer et al., 2007, for similar evidence using a cognitive load paradigm). A common feature of the studies that report attentional modulation of emotional processing is that the non-emotional task usually involves a high attentional load; therefore, sufficient attentional resources were not available to process the emotional content of the stimuli (Lavie, 1995; Pessoa et al., 2002, 2005; Okon-Singer et al., 2007; Palermo and Rhodes, 2007).

The present study is a follow-up of the Sassi et al.’s (2014) study, although only two tasks were used: the emotional task, in which subjects attended to the emotional expression of the face, and the shape task, in which subjects attended to the shape of the glasses so that the emotional facial expression was task-irrelevant. In addition, whereas many studies have investigated the processing of threatening stimuli using fearful faces, we were interested in extending our affective priming studies to other negative emotional expression. Thus, anger faces were selected for the present study. Anger is frequently exhibited in daily life as much as other negative expressions such as fear and sadness, but few studies have used this emotional expression in paradigms that used attentional manipulations.

On the basis of our previous results, we expected an affective priming effect with the emotion task, but not with the shape task. However, as Okon-Singer et al. (2007) pointed out, it is necessary to dissociate attention-dependent processing from automatic processing (at least the “weak” notion of automaticity, Tzelgov, 1997; Pessoa, 2005). Despite the lack of behavioral priming effects, which might depend on the availability of attentional resources, is still possible that processing of the negative facial expression in the shape task occurs in a “strong” automatic way, independently of both attentional resources and task relevance. Negative facial emotional expressions may be related to threat and therefore they may be behavioral relevant stimuli that require a fast automatic reaction to foster survival. If that were the case, we would be able to detect emotion-related brain activation even when subjects’ top–down attention is allocated to an emotion-irrelevant feature of the face prime that requires fine-grained discrimination (the shape task). The rational for that hypothesis is the existence of a neural circuitry comprising both subcortical and cortical areas, that is involved in the rapid and automatic detection of threatening salient stimuli, and that may play a crucial role for survival (Vuilleumier, 2005).

In the study, we carried out distributed source analyses (Fuchs et al., 1999) over the ERP generated by the face. Unlike dipole analysis (Scherg and Von Cramon, 1985), which uses very few sources and needs strong *a priori* hypotheses about their characteristics, the source analysis technique represents the cortical brain activity through the intensity of a large number of cortical generators, providing a more realistic simulation of brain functioning. Among the several approaches available to solve the inverse problem of reconstructing the cortical sources that generated the recorded scalp potentials, we opted for a well-established post-processing method (Inuggi et al., 2010, 2011a,b; Gonzalez-Rosa et al., 2013). It employs a sLORETA-weighted accurate minimum norm method (SWARM) algorithm (Wagner et al., 2007), which allows for the low reconstruction error of sLoreta (Pascual-Marqui, 2002) and also outputs a current density vector field that can later be post-processed.

We focused then on the cortical areas that are involved in the processing of the fine-grained facial features. Briefly, the process of recognizing the static (identity, gender, familiarity) and the dynamic (emotional expressions and gaze direction) characteristics of the observed face are thought to rely mainly on a cortical stream (Haxby et al., 2000; Palermo and Rhodes, 2007) embracing both the classical ventral stream (Ishai et al., 1999) and the superior temporal sulcus (STS). The ventral stream originates in the occipital areas and propagates through the occipital face area (OFA) and the fusiform face area (FFA). The FFA is specialized in decoding fine-grained static facial characteristics (Kanwisher et al., 1997; Halgren et al., 2000; Holmes et al., 2003; Bayle and Taylor, 2010), while the STS, especially its posterior part (pSTS), is involved in the processing of dynamic facial features, such as eye gaze, and in decoding the emotional information from facial features (Puce et al., 1998; Allison et al., 2000; Hoffman and Haxby, 2000; Said et al., 2010). Previous studies have observed that that FFA activates more with facial than with non-facial objects (see Haxby et al., 2000, for review), and therefore we expected reduced activation of that area in the shape task (focused on a non-face feature) compared to the emotion task (focused on a facial feature).

To model the activation of these areas, we performed both sources and sensors analysis in correspondence to the main ERP components. Besides modeling the two mostly investigated early components, the posterior P1 and the lateral occipito-temporal N170, we also modeled the anterior N1 (Luo et al., 2010) and a later positive component, peaking around 230–250 ms, whose name and temporal location vary greatly across studies (e.g., VPP in Luo et al., 2010; P270 in Liu et al., 2012). Both P1 and N1 components have been associated with a first stage of automatic processing that differentiates negative facial expressions from positive or neutral facial expressions (Pourtois et al., 2004; Luo et al., 2010), which reflects an early negativity bias (Smith et al., 2003). The N170 component has been involved in the distinction between faces and non-faces stimuli (Bentin et al., 1996; Rossion et al., 2003; Itier and Taylor, 2004; Luo et al., 2010). As the aforementioned components have been shown to be affected by affective processing in an early phase of perception and attention processing (Carretié et al., 2004; Eimer and Holmes, 2007; Luo et al., 2010), they constitute the main goal of our analysis of the first 300 ms postface prime onset.

Source analyses were also employed to assess whether angry and non-angry (happy and neutral) expressions were processed differently when attention was directed to emotion-irrelevant facial features. Specifically, because negative emotional expressions are behavioral relevant stimuli, we expect activation in the ventral attention network (VAN), which is supposed to detect behavioral relevant but task-irrelevant stimuli and to exert a bottom–up modulation over the dorsal attention network (DAN; Corbetta and Shulman, 2002; Corbetta et al., 2008). However, because emotion-relevant and emotion-irrelevant features were foveally presented, we did not expect any reorienting process by the DAN, which is responsible for top–down control as it contains, specifically in the Frontal Eye Field region, the proper circuitry to moves the eyes to the selected target. Thus, we may be able to test the hypothesis that the VAN might activate independently from the DAN by assessing brain activity in both the temporo–parietal junction (TPJ; VAN) and the intra-parietal sulcus (DAN). These networks are considered supramodal (Macaluso et al., 2002; Green et al., 2011) and not directly related to face processing. Because their involvement in bottom–up and top–down control is derived mainly from functional magnetic resonance imaging (fMRI) studies, whose temporal resolution is not enough as to be coupled with electroencephalography (EEG) activation findings, their activation time course will be investigated here in the temporal proximity of the classical ERP peaks, where the face feature decoding process is expected to occur.

## MATERIALS AND METHODS

### SUBJECTS

Twenty-eight healthy, young (mean age 22.1 ± 2.3 years, range 19–30) subjects with no history of neurologic or neuropsychiatric disorders were recruited to participate in this study. Fourteen subjects (11 females and 3 males) participated in each task condition (emotion and shape). All subjects were right-handed according to their self-report and gave their written informed consent for participation in the study.

### TASK

**FIGURE 1:**
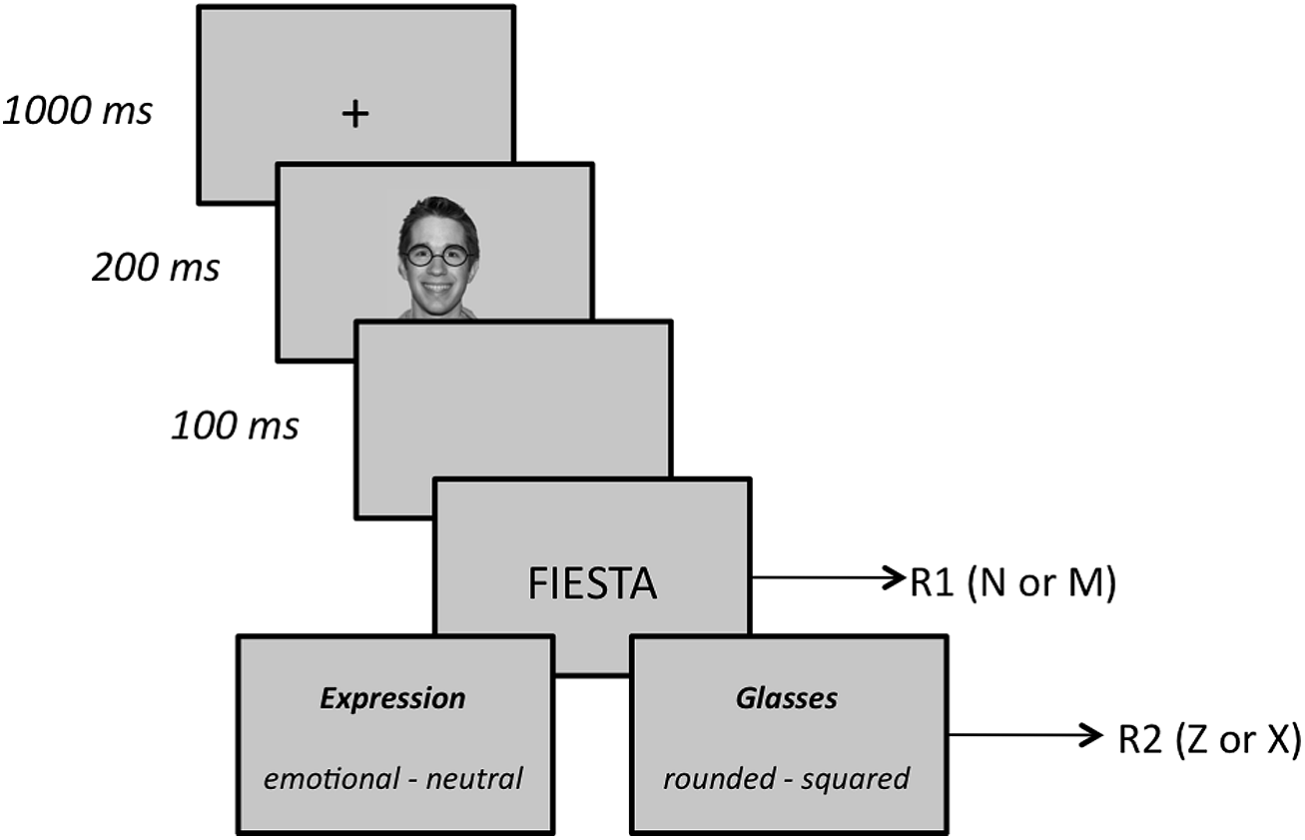
**Sequence of events and time duration in the experiment**.

Subjects were tested individually in a sound-attenuated room. A computer program generated by E-Prime 2 (Schneider et al., 2002) controlled the experiment. The stimuli were presented on a 17^′′^ TFT monitor (screen resolution: 1024 by 768 pixels; background color: silver – RGB: 200, 200, 200) and participants responded via the keyboard. We used three grayscale pictures (4.5 cm wide by 7.7 cm height) of human faces as prime stimuli, one for each facial expression (happy, angry, and neutral). These stimuli were taken from the NimStim Set of Facial Expressions (Tottenham et al., 2009; the reference codes of the selected faces are 20_M_HA_O, 20_M_NE_C, and 20_M_AN_O). By using photo-editing software, we created two versions of each picture, one wearing rounded glasses, and the other wearing squared glasses. As target stimuli, we used 36 Spanish words divided into two sets, one comprising 18 positive words, the other containing 18 negative words. Mean valence ratings for the words of the two sets ranged from 1.7 to 2.8 (*M* = 2.3) for positive words and from –0.9 to –1.8 (*M* = –2.3) for negative words, according to a preliminary study (*N* = 124; scale ranging from –3 to +3; see Sassi et al., 2014). Positive and negative words were matched for word frequency, familiarity, and word length using the LEXESP database (Sebastián-Gallés et al., 2000). Each trial consisted of the following sequence (the trial scheme is summarized in **Figure 1**). First, a 1000-ms fixation point (a plus sign) appeared in the center of the screen followed by one of the three prime faces, which was presented for 200 ms. Then, after an interval of 100 ms (stimulus onset asynchrony, SOA = 300 ms), a target word was shown (in capital letters and black font) and subjects indicated whether the word was positive or negative by pressing the “n” or “m” key on the computer keyboard as quickly and accurately as possible (this first response is referred to as R1). Both prime faces and target words were presented centered. The specific response-key mapping was counterbalanced across participants. Immediately following R1, a double-choice question appeared on the screen, and subjects were prompted to press, with no time limit, the key (“z” or “x”) that corresponded to the correct answer (hereafter, R2). In the emotion condition, subjects were asked whether the prime face was neutral or emotional (the emotion task), whereas in the glasses’ shape condition they were asked whether the face wore rounded or squared glasses (the shape task). The whole experiment included 72 congruent trials, 72 incongruent trials, and 144 neutral trials. In congruent trials, the prime face and the target word belonged to the same affective valence, either positive, as it happened in happy-positive trials (*N* = 36) or negative, as it happened in anger-negative trials (*N* = 36). In incongruent trials, a prime face with different valence preceded the target word, as it happened in happy-negative trials (*N*= 36) and anger-positive trials (*N* = 36). In neutral trials, a neutral prime face preceded the target word, as it happened in neutral-positive (*N* = 72) and neutral-negative (*N* = 72) trials. Target words were drawn from each set at random, with the constraint that each word appeared in two congruent trials, in two incongruent trials and in four neutral trials. A short practice block of 18 trials preceded the experimental trials.

### EEG RECORDINGS AND PREPROCESSING

Electroencephalography was recorded using 59 scalp channels mounted onto an elastic cap (ActiveCap, Brain Products GmbH), according to the 10–20 international system, with the reference located close to the vertex. The EEG signal was amplified (BrainAmp, Brain Products GmbH), digitized (1000 Hz sampling frequency), and filtered (0.1 to 40 Hz). The electrode impedance was kept below 5 KΩ. Four additional electrodes were placed to monitor the left/right and horizontal/vertical ocular activity. The eye movements’ artifacts were corrected with an independent component analysis (ICA) Ocular Artifact Reduction algorithm (Vision Analyzer, Brain Products GmbH). The ERPs were obtained by averaging the EEG epochs from –250 to +300 ms with respect to face onset, using the first 200 ms for baseline correction. Data were finally re-referenced using a common average reference approach.

### ERP COMPONENTS DEFINITION

According to previous studies, we focused on the P1 and N170 components and also on N1, which peaks in frontal regions at ∼100 ms. Additionally, our data revealed a late positive deflection, peaking at ∼240 ms, that was also investigated. Four pairs of sensors clusters, whose amplitude was calculated as the mean amplitude of their constituent sensors, were defined to model the ERP components. In each subject and for each experimental condition, the amplitudes of components’ peaks were calculated as the maximum positive/negative deflections within the time windows specified in **Table 1**. To better compare ERP results with source analysis results, a further cluster, conventionally not investigated in previous studies, was defined for the N170 period that covered the temporo–parietal region. These eight cluster measures were subjected to statistical analyses. In further analysis, the two occipital clusters were merged into a single cluster, and its activation was expressed in terms of the lateralization of its medial–lateral center of gravity, calculated with the following formula:

COGX=(a*PO8+b*PO4+c*O2−a*PO7−b*PO3−c*O1)/2*(a+b+c)

where a,b,c represent the medial–lateral coordinates of those electrodes in a 10–20 extended system.

**Table 1:** Event-related potential components investigated, electrodes contained in the eight clusters used, and the window of interest used to define the component’s peak.

Components	Cluster name	Electrodes in cluster	Window of interest
N1	L/R Frontal	F3/4, FC3/FC4	80–130
P1	L/R Occipital	PO7/8, PO3/4, O1/2, Oz, POz	
N170	L/R Occipito-temporal	PO7/8, PO3/4, P7/8	130–190
N170	L/R Temporo–parietal	P5/6, CP5/6	
P240	All the previously defined clusters		220–260

### SOURCE ANALYSIS

A preliminary ICA (Hyvarinen, 1999) was performed on ERP data, which allowed for the decomposition of the signal to noise-normalized independent components (ICs). Only those ICs that showed an SNR below 1 across all intervals of interest (from –250 to 300 ms with respect to the facial onset) were removed from the ERP data (Inuggi et al., 2011a,b). The source activity was reconstructed using the cortical current density (CCD) model with a conductor volume defined by a 3-compartment boundary element method (BEM), with conductivity values of 0.33-0.0042-0.33 S/m (Fuchs et al., 2002), derived from the FSL MNI template (www.fmrib.ox.ac.uk/fsl), dimensions of 91×109×91 and a voxel size of 2×2×2 mm. The sources number (6899) and positions were obtained by sampling the cortex (5 mm wide), with their orientations fixed perpendicular to the cortical patch they originated from, and their intensities were calculated using the SWARM algorithm (Wagner et al., 2007). The CCD was reconstructed with the Curry V6 software (Neuroscan Inc., Herndon, VA, USA).

#### ROI definition

Cortical activity was calculated in seven pairs of right and left regions of interest (ROI) involving lateral fusiform gyrus (BA37), posterior superior temporal sulcus (pSTS), TPJ plus inferior parietal lobule (TPJ+IPL), intraparietal sulcus (IPS), middle frontal gyrus (MFC), inferior frontal gyrus (IFG), and primary visual area (V1). In an additional analysis, the two V1 ROIs were merged into a single ROI, and its activation was expressed in terms of lateralization of its medial–lateral center of gravity, calculated as is explained later on.

**FIGURE 2:**
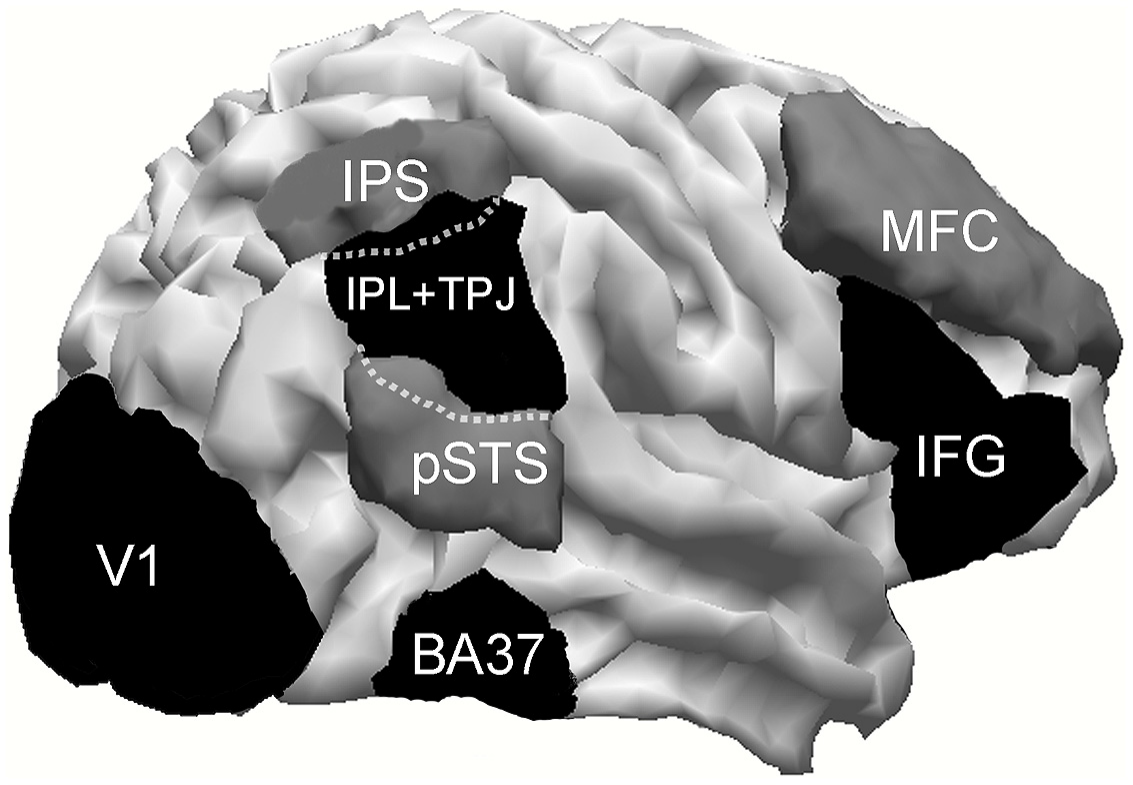
**Cortical areas investigated.** IPS, intraparietal sulcus; pSTC, posterior superior temporal sulcus; TPJ, temporo–parietal junction; BA37, lateral temporo–occipital cortex; MFC, middle frontal cortex; IFG, inferior frontal gyrus. Within dotted lines, is represented that part of TPJ which overlaps with IPS and pSTS.

Regions of interest were manually drawn on the MRI images using the Curry software internal anatomical atlas and previous research as references. TPJ+IPL ROI was created starting with the strict TPJ definition of Mort et al. (2003) but also included the inferior parietal lobe, like most studies that investigate the VAN and that located their activations around these areas. Its resulting center of gravity will clarify more specifically the anatomical localization of this activation. To take into account possible between-subjects electrodes’ slight montage misallocation, ROIs were enlarged (5 mm wide) and then smoothed (2 mm wide). ROIs are illustrated in **Figure 2**.

#### ROI activity

Three periods were investigated: L100, where N1 and P1 are active, L170, which corresponds to the N170 peak, and L240, which corresponds to our late peak. Within these periods, the mean cortical activation of each ROI was separately calculated using the following procedure: (i) within each latency, the intensity of all of the active sources contained in the ROI were summed; (ii) the latency with the highest value was defined as the peak latency (PL); and (iii) a 40 ms-length temporal window, centered on that peak, was used to calculate the area total activity (TA) within each period of each area, as was previously described (Inuggi et al., 2011a; Gonzalez-Rosa et al., 2013). This procedure was performed separately for each ROI, thus allowing us to take into account the onset differences of nearly simultaneous components (e.g., P1 and N1) and to create periods of the same temporal length to ensure proper comparisons. The length of the time window was selected according to a previous study (Gonzalez-Rosa et al., 2013).

The activations of center of gravity, decomposed in the medial–lateral (CX), anterior-posterior (CY), and ventral-dorsal (CZ) positions, were calculated using the following formula (e.g., CX):

$$CX=\left(\Sigma_{ij}S_{ij}*X_{ij}\right)/\Sigma_{ij}S_{ij},$$

where s_ij_ is the intensity of the i-th source at timepoint j and X_ij_ is the medial–lateral position of the i-th source at timepoint j.

### STATISTICAL ANALYSIS

The effects of the between-subjects factor *task type* (emotion task, shape task) and the within-subjects factor *face expression* (angry, happy, or neutral) and *hemisphere* (left and right) over TA within each area and period were analyzed with a mixed analysis of variance (ANOVA). The Kolmogorov–Smirnov test was used to examine the normal distribution of the data, and, when appropriate, the Greenhouse–Geisser correction was applied. The significance level of the main effects (task type, emotional expression, and hemisphere) and their interactions were corrected for multiple comparisons (14 ROI × 3 periods) using a false discovery rate (FDR) approach, but using a more conservative version (Benjamini and Yekutieli, 2001) compared to standard FDR. According to its formula (α/Σ_i=1..k_(1/i), where i = 42 is the number of multiple comparisons and α = 0.05 is the predetermined *p*-value), we report only the significant *p*-values below 0.0112. Because the number of multiple comparisons was lower in the ERP analysis (8 cluster × 3 periods), the corrected threshold was 0.0132. The size effects were reported through the $\eta_{p}^{2}$ value. *Post hoc* comparisons of within-subjects (facial expression) and between-subjects (task type) factors were performed with paired and unpaired *t*-tests. The multiple pairwise comparisons of facial expressions were adjusted with the Bonferroni correction.

To provide the ERP equivalent of our source analysis results, a mixed ANOVA, analyzing the effects of *task type* and *face expression*, was also performed over the ERP electrode clusters that overlay the ROI of the sources significantly affected by our experimental factors.

## RESULTS

### BEHAVIORAL DATA

Trials with incorrect responses to the target word (R1; 1.8 and 1.5% for the emotion task and the shape task, respectively), and trials with incorrect responses to the to-be-attended facial feature (R2; 3.1 and 5.0% for the emotion task and the shape task, respectively) were excluded from analysis. In addition, we excluded trials with RTs below 200 ms (anticipations) or more than three standard deviation (omissions) from the subject’s mean for each condition (1,90%). The mean RT for R1 in the emotion task was 790 ms (SD = 144) for congruent trials (happy face/positive word and angry face/negative word trials) and 825 ms (SD = 164) for incongruent trials (angry face/positive word and happy face/negative word trials). In the shape task, the mean RT was 767 ms (SD = 173) for congruent trials and 768 ms (SD = 155) for incongruent trials. These means were submitted to mixed ANOVA with *congruency* (congruent, incongruent) and *task type* (emotion, shape) as factors. There was a main effect of *congruency*, *F*(1,26) = 9.75; *MSE* = 464; *p* = 0.004; $\eta_{p}^{2}$ = 0.27, revealing that responses were faster for congruent than for incongruent trials (this difference represents the affective priming effect, *M* = 18 ms). However, this effect was qualified by a *congruency* by *task type* interaction, *F*(1,26) = 9.10, *MSE* = 464, *p* = 0.006, $\eta_{p}^{2}$= 0.26. *Post hoc* Fisher’s least significant difference (LSD) tests (*MSE* = 25411, *df* = 26,479) revealed significant *congruency* effect for the emotion task (priming effect = 35 ms, *p* < 0.001) but no effect at all for the shape task (priming effect = 0.6 ms, *p* = 0.941). Results, thus, replicate those obtained in our previous behavioral study (Sassi et al., 2014). To support the goodness of our protocol, we verified that neither the main effects of *word valence*, *F*(1,26) = 1.56, *p* = 0.222, and *task type*, *F* < 1, nor their interaction, *F*(1,26) = 1.30, *p* = 0.26, were statistically significant with the neutral expression. Analysis of error rate (CR1) revealed no statistically significant effects.

### SOURCE ANALYSIS DATA

**FIGURE 3:**
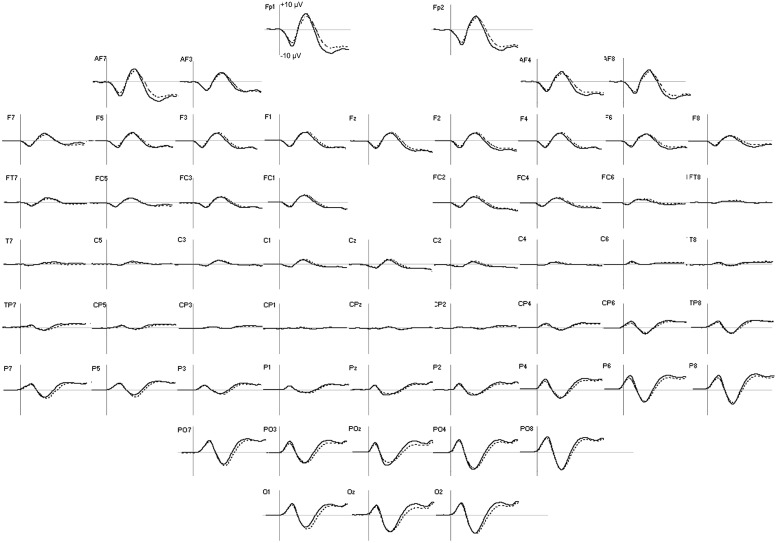
**Group averages of ERP in emotion (solid line) and shape (dotted line) tasks in the first 300 ms after facial stimulus presentation.** For all the electrodes, the vertical scale boundary is set at +10 μV.

The group averages of the evoked potentials elicited by the two tasks, which merged the three emotional faces, are displayed in **Figure 3**. **Table 2** summarizes the activation’s center of gravity coordinates and PL values of the ROIs, where a significant effect of either task type or face emotion could be observed.

**Table 2:** Talairach coordinates of activations; center-of-gravity in right IPL + TPJ ROI at L170.

Area	Task	Neutral			Happy			Angry		
		X	Y	Z	X	Y	Z	X	Y	Z
R TPJ 170	Emotion	49	–51	24	48	–49	23	49	–49	22
	Shape	49	–51	29	48	–53	28	49	–50	23

#### Effect of task type

**FIGURE 4:**
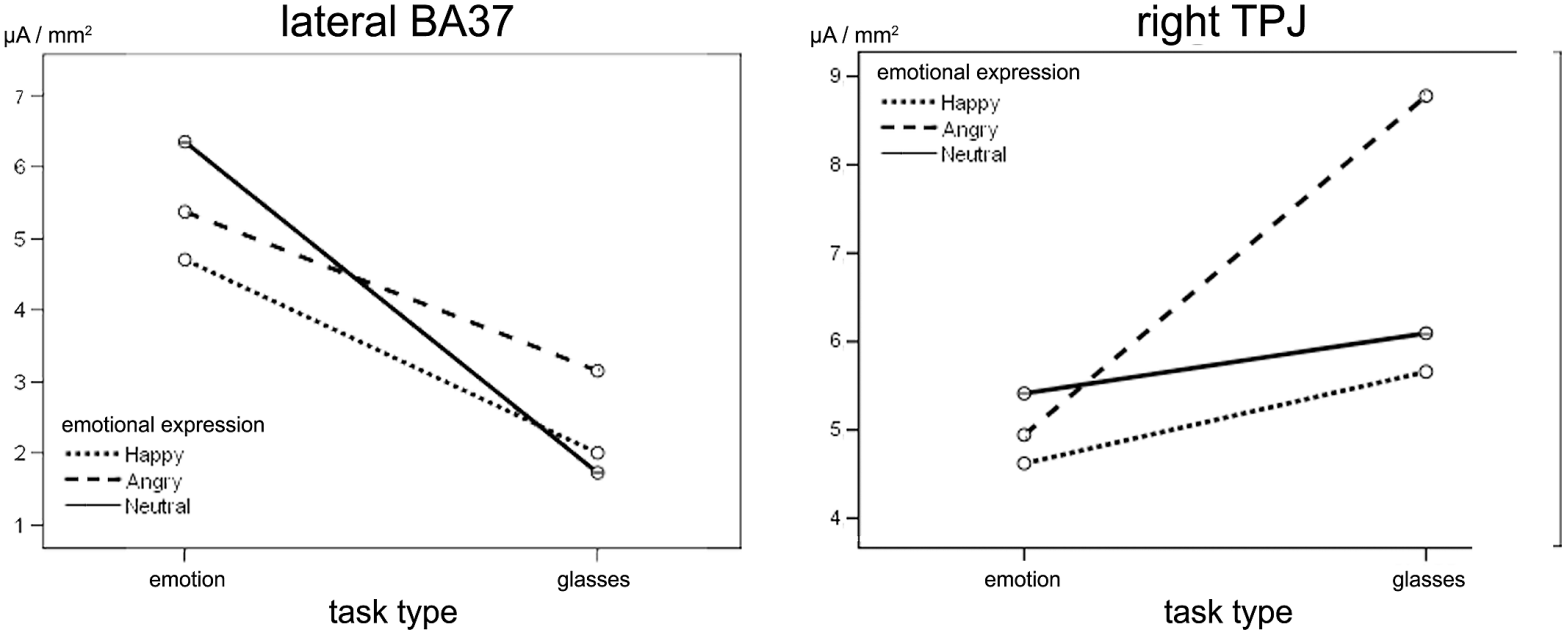
**Effect of emotional expression and task type at L170. (Left)** Task type effects on right lateral BA37. No significant differences were observed for facial emotion. **(Right)** Right TPJ sensitivities to angry facial expression in shape task only. On the y-axis, the mean activity of each ROI in the L170 time window is expressed in μA/mm^2^.

During L170, an effect of task type was observed in lateral BA37 activity, [*F*(1,26) = 7.93; *p* = 0.011, $\eta_{p}^{2}$ = 0.26], which was less intense (**Figure 4**) in the shape task (*M* = 2.28, SD = 0.6 μA/mm^2^) than in the emotion task (*M* = 5.4, SD = 0.5 μA/mm^2^).

#### Interaction between task type and facial expressions

**FIGURE 5:**
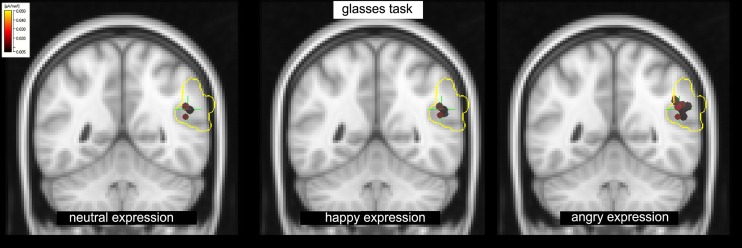
**The shape task: increased activation in response to the angry facial expression (right) compared to happy (center) and neutral (left) expressions in the TPJ within the IPL+TPJ ROI (voxels enclosed within the yellow borders) at L170**.

A significant type task x facial expression × hemisphere interaction was observed in IPL+TPJ during L170, [*F*(1.518,39.45) = 6.41, *p*= 0.010, $\eta_{p}^{2}$ = 0.23]. *Post hoc* analyses revealed that the *type task* × *facial expression* was significant only for the right side, [*F*(1.81,37.06) = 5.35, *p*= 0.010, $\eta_{p}^{2}$ = 0.218]. Additionally, while facial expressions did not differ from each other in the emotion task, an effect of facial expression was observed in the shape task when facial expressions had to be ignored, [*F*(1.58,20.56) = 12.06, *p*= 0.001, $\eta_{p}^{2}$ = 0.48], with higher activation to angry facial expressions (*M* = 8.1, SD = 1.1 μA/mm^2^) compared to both happy (*M* = 5.8, SD = 0.9 μA/mm^2^, *p*= 0.002) and neutral (*M* = 6.1, SD = 0.8 μA/mm^2^, *p*= 0.002) ones (**Figure 4**, right; **Figure 5**). The center of gravity position of cortical activation in IPL+TPJ ROI, reported in **Table 2**, was located in close proximity to the TPJ defined by Mort et al. (2003), as shown in **Figure 5**. We thus will refer to it as TPJ activation. No modulation over the IPS, pSTS, or middle and inferior frontal areas were observed at any latency.

#### Lateralization of visual area activity

**FIGURE 6:**
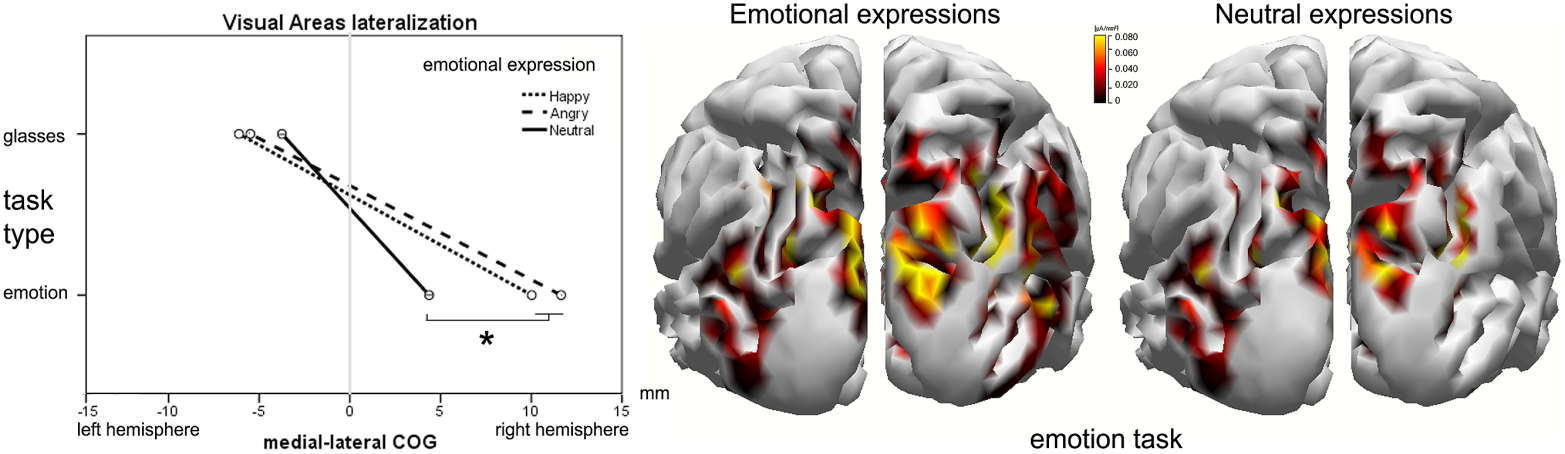
**Visual area lateralization around L100. (Left)** The effect of facial expression and task type on the medial–lateral position of the activation’s center-of-gravity (COG). The cortical current density (CCD) results of the emotional facial expression (center) compared to the neutral facial expression **(right)** in the emotion task. On the y-axis, the mean activity of the ROI in the L100 time window is expressed in μA/mm^2^.

During the P100 component, the medial–lateral center of gravity (CX) of the visual areas was more lateralized to the right hemisphere in the emotion (*M* = –5, SD = 0.9 mm) task compared to the shape (*M* = 8.7, SD = 2.1 mm) task [*F*(1,26) = 8.21, *p*= 0.010, $\eta_{p}^{2}$ = 0.281; **Figure 6**]. A significant task type × facial expression interaction was observed in visual areas, [*F*(1.53,38.21) = 6.55, *p*= 0.010, $\eta_{p}^{2}$ = 0.18]. The effect of facial emotion on the activation lateralization was observed only in the emotion task, with the angry (*M* = 12, SD = 2.5 mm, *p*= 0.011) and happy (*M* = 10, SD = 2.2 mm, *p*= 0.010) faces more lateralized to the right hemisphere with respect to the neutral faces (*M* = 4.7, SD = 2 mm). No significant differences emerged in the L240 interval.

### ERP DATA

**FIGURE 7:**
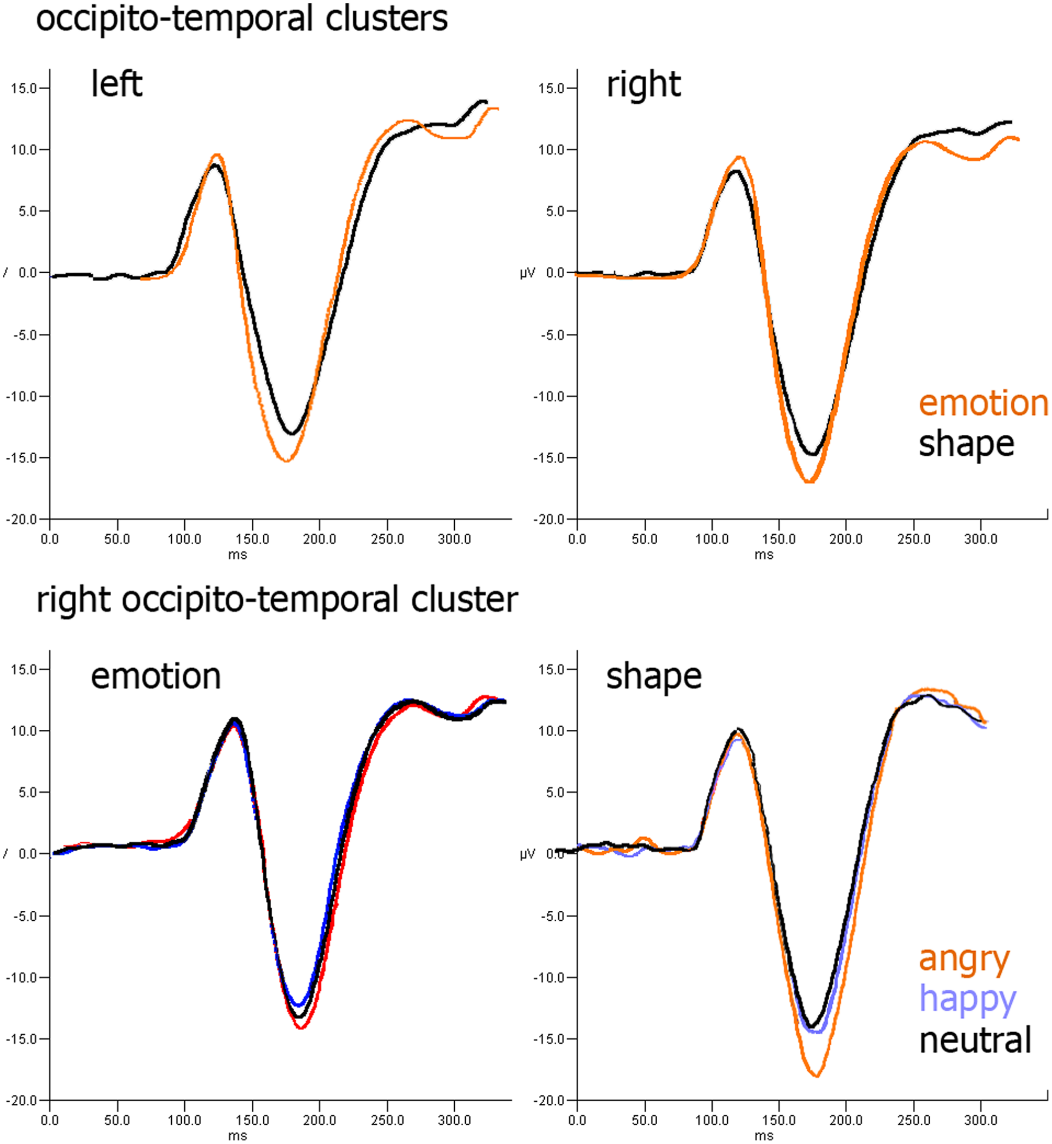
**ERP results: (upper row) effect of task type over occipito-parietal cluster; (lower row) effect of facial expression over occipito-parietal cluster in emotion (left) and shape (right) tasks**.

During the P100 component, the medial–lateral center of gravity of the cluster obtained by merging the right and left occipital clusters was modulated by task type [*F*(1,26) = 5.45, *p*= 0.011, $\eta_{p}^{2}$ = 0.25], which was more right-lateralized in the emotion task (*M* = 11.3, SD = 4.5 mm), than in the shape task (*M*= –0.9, SD = 3.8 mm). At ∼170 ms, the occipito-temporal cluster that overlays the lateral BA37 was not affected by the task type. In the right occipito-temporal cluster, which should provide the ERP equivalent of the right TPJ activation, a significant interaction was found between task type and facial expression in the occipito-temporal cluster [*F*(1.52,21.13) = 5.20, *p*= 0.012, $\eta_{p}^{2}$ = 0.24]. Nevertheless, we found a trend (*p*= 0.065) versus a more negative peak to angry faces compared to neutral ones in the shape task (**Figure 7**). No differences emerged within the parieto-temporal cluster.

## DISCUSSION

In this study, the effect of a fine-grained, emotion-irrelevant, discriminatory task on the early emotional faces processing was investigated by reconstructing the cortical generators of the scalp-recorded potentials. Our main objective was to evaluate if angry expressions were processed differently from non-angry (neutral and positive) expressions when attention was diverted to another task. We opted to engage subjects in a fine discrimination of the shape of the glasses worn by the face stimuli, a task that was supposed to deplete, according to our previous behavioral study, the attentional resources (see Sassi et al., 2014). Several previous studies assessed the interaction of attention and emotion when emotion-relevant and emotion-irrelevant stimuli did not share the same geometrical space (Vuilleumier et al., 2001; Holmes et al., 2003). Because the redirection of the subject’s attention to another position may represent a potential confounding issue, we opted to place both the emotion-relevant and emotion-irrelevant features in the same foveal position, removing any obstacles to the automatic processing of emotional faces when asked to ignore them. In addition to investigating the peculiar processing of ignored angry faces, we were also interested in giving a neurophysiological explanation for the loss of the affective priming effect observed in our behavioral results (Sassi et al., 2014, current study) when subjects were involved in an emotion-irrelevant task. We concentrated our analysis on the cortical areas involved in the processing of the fine-grained facial features, which are supposed to be highly modulated in a top–down manner by the observers’ attention, making its processing not pre-attentive but strictly related to the availability of attentional resources. Moreover, considering the high priority of aversive facial expressions in capturing attentional resources, we also focused on the parietal areas that belong to both the ventral (TPJ) and the dorsal (IPS) attention networks and the partially overlapped frontal areas of the two networks, the inferior (IFG) and middle (MFG) frontal gyri (Fox et al., 2006).

### THE EFFECT OF ATTENTION ON THE VENTRAL STREAM

In the present study, we confirm that the ventral stream is highly modulated by the observer’s attention. The activity of the occipital areas at ∼100 ms was more right lateralized in the emotion task than in the shape task and, more notably, when subjects attended to the facial expression, activation produced by emotional face expressions was more right lateralized than activation produced by neutral faces. Such selectivity disappeared when subjects attended to the glasses’ shape.

Considering that the assessment of FFA activity through scalp recordings is widely questioned, as the area lies within the inferior part of the temporal cortex, we created the lateral BA37 ROI because previous neuroimaging studies showed a correlation between the N170 EEG component, calculated by electrodes overlaying it and fMRI-derived FFA activity (Horovitz et al., 2004; Sadeh et al., 2010), which suggests that surface electrodes may capture at least part of FFA activity. Additionally, electro-corticography studies have revealed that lateral BA37 is also involved in face processing (Rossion et al., 2003; Tsuchiya et al., 2008). At ∼170 ms, lateral BA37 activation was reduced in the shape task compared with the emotional task, which suggests that when subjects were asked to ignore the facial expression and just concentrate on the glasses’ shape, the detailed face features might not have been very distinctive. This result agrees with previous findings that report larger activity in FFA for faces compared to non-face objects (Haxby et al., 2000; Rossion et al., 2003). Taken together, our behavioral and neurophysiological results strongly suggest that our shape task succeeded in guiding subjects’ attention away from any face feature, preventing any conscious monitoring of the emotional content of the face. In the long debate over the pre-attentive automaticity of emotional processing, our results suggest that an appropriate level of attention is needed to process emotional expressions. Although presented in the same visual focus, the reduced BA37 activity and the loss of emotional selectivity of primary visual areas in the shape task suggest that subjects presumably focused their attention just on the glasses’ shape and ignored the underlying emotional expression.

The lateralization of the activations found in the present study deserves further comments. The lateralization of emotional processing is still an open issue because the two main theories, supporting either the right-hemisphere hypothesis (RHH; Borod et al., 1998; Bourne, 2010) or the valence-specific hypothesis (VSH; Mandal et al., 1991; Adolphs et al., 2001), have been questioned by more recent fMRI meta-analysis investigations (Fusar-Poli et al., 2009; Sabatinelli et al., 2011). The bulk of evidence shows bilateral activation for emotional face processing in most emotion-related areas, although lateralization might be modulated by gender (see for example Wager et al., 2003). In the present study most (22 out of 28) of the subjects were women, and our data are consistent with a previous EEG report specifically investigating the gender effect over emotional face processing. Proverbio et al. (2006) in fact found maximal P1 amplitude over the right occipital cortex in both genders, consistently with our results showing that in the emotion task occipital activity around 100 ms was right lateralized. The lack of a right lateralization observed in our data during the N170 may appear inconsistent with the widely accepted right predominance of FFA in face processing (Kanwisher and Yovel, 2006). However, this again agrees with Proverbio et al.’s (2006) findings of a right lateralization of N170 only in men. In contrast, women exhibited a bilateral pattern. These results can help foster better understanding of the inconsistencies in the literature on the right hemisphere advantage in the occipito-temporal cortices when processing faces and confirm the relevance of incorporating gender information.

### ANGRY FACIAL EXPRESSION PROCESSING

Although both static and emotional features appeared under-processed by canonical face processing cortical areas, unattended angry expressions were able to activate the TPJ, a cortical expanse implicated in a wide spectrum of high-order cognitive functions ranging from social cognition (Saxe and Kanwisher, 2003) to attention selection (Corbetta and Shulman, 2002). The latter branch of investigation showed that the TPJ is part of the VAN, a fronto-parietal network that, during focused activities, is formally involved in re-orienting (shifting) attention to stimuli relevant to the immediate goal. Nevertheless, because the attentional focus covered a similar area in both tasks, no reorienting process was expected, as our IPS activity also indicates. The latter is in fact part of the DAN, which contains the proper circuitry to implement the focus reorienting, and was not modulated by our experimental conditions. The absence of any modulation over frontal areas might be interpreted accordingly; the integration between ventral and DANs, needed for attention re-orienting, occurs in such aforementioned frontal areas where the two networks highly overlap (Fox et al., 2006).

Thus, the present findings support the proposal that VAN activation, at least in its parietal areas, might not exclusively be involved in attentional reorienting. It is consistent with more recent reports that suggest that TPJ activity might be triggered by both external sensory stimuli and internal memory-based information, thus providing bottom–up signals to other systems about relevant stimuli for further inspection (Cabeza et al., 2012). In agreement with the present results, VAN activity has also been observed when behaviorally relevant, rather than salient, stimuli are presented while the individual is engaged in another task (Corbetta et al., 2008). Accordingly, the activation of TPJ just when the unattended face was shown with an angry expression suggests that negative emotions can pre-attentively evoke bottom–up cortical signals, according to their behavioral relevance, even when attention is focused on emotion-irrelevant features in a task that we assumed exhausted the attentional resources to process the emotional content of faces. Because the ventral stream and STS were not modulated by the degree of unattended emotional content and the VAN is considered a supramodal network (Macaluso et al., 2002; Green et al., 2011) not able to decode the threatening pattern from facial expression, we suggest that TPJ activation might be triggered from other brain regions. Several neuroimaging studies suggested that, in parallel with the cortical stream (Palermo and Rhodes, 2007), a subcortical pathway, that reaches the amygdala through fast and coarse subcortical inputs that originate in the superior colliculus and finally project onto fronto-parietal areas, is thought to implement a brain circuitry specialized in emotional attention (Vuilleumier, 2005). This circuitry, likely partly modulated by the attentional focus (Pourtois et al., 2013) is involved in the rapid and automatic detection of negative facial expressions (for review; see Vuilleumier and Pourtois, 2007), and it seems to play a crucial role in directing attention and information processing to threatening stimuli (Ohman and Mineka, 2001). Because reconstructing amygdala activity with EEG presents several accuracy limitations, as it will be discussed later, further studies that integrate EEG with neuroimaging techniques are surely needed, but our data are consistent with such a model. A previous MEG study showed in fact that the amygdala activates as early as 100 ms after stimulus presentation (Streit et al., 2003), a latency early enough to trigger TPJ activation at ∼150–170 ms. The present TPJ activation of ∼170 ms is consistent with a recent ERP study that investigates the threat detection advantage (Feldmann-Wüstefeld et al., 2011), which revealed that angry and happy expression processing started to differ at ∼160 ms. This suggests that angry faces may trigger a fear module that enables their rapid processing and recruit additional attentional resources, possibly by means of TPJ, as is here hypothesized.

In conclusion, within the VAN, TPJ activation at this early latency primarily signals the behavioral relevance of a task-irrelevant aversive stimulus, irrespective of whether that stimulus requires a physical shift of attention (involving the dorsal network). The fact that such a trigger was not followed by an actual over-processing of face features is likely due to the task demands that, immediately after face offset (∼200 ms), required that subjects focus on word onset and the corresponding response related to its emotional valence.

### DIFFERENCES BETWEEN SOURCES AND SENSORS ANALYSIS

In the present paper, we aimed to provide an ERP-equivalent of the activations produced by source analysis. We thus focused this analysis only on the time windows and clusters that surround the cortical areas affected by our experimental conditions. ERP analysis found that attended emotions, compared to ignored emotions, have their occipital P1 peak more right lateralized but was unable to assess the selectivity toward attended emotional faces, which disappeared in the shape task. In a similar manner, ERP analysis could detect the interaction between task and emotions at ∼170 ms in the right occipito-temporal cluster, but it did not find a significant difference between angry and non-angry ignored faces. Of course, the current ERP approach is only one of many possible approaches. We are not concluding that another ERP analysis would have been unable to locate the same effects found with source analysis. However, even if such an effect had been encountered in a cluster or in a channel (e.g., CP4 or CP6), it would have been impossible to clearly attribute it to one of the areas beneath and close to the sensors cluster. Ideally, both pSTS and BA37 would have been valid candidates, and we could have argued that because they are part of the cortical stream supposedly deputed to extract face features, they would have presumably shown such functioning also in the attended condition, but that doubt would have persisted, and the involvement of TPJ could have been just one of the possible hypotheses. Instead, source analysis, when calculating the center of gravity of the large ROI covering the temporal and parietal lobe, indicated the TPJ involvement.

### METHODOLOGICAL CONSIDERATIONS AND LIMITS OF THE PRESENT INVESTIGATION

The main limits of EEG source analysis are its high sensitivity to artifacts, the low signal-to-noise ratio and the limited spatial resolution. To properly address these limits, we employed a consolidated methodological approach (Inuggi et al., 2010, 2011a,b; Gonzalez-Rosa et al., 2013), which has consistently proved to obtain results in line with the neuroimaging literature. We used a seed-based analysis instead of a voxel-wise one because this approach is often used in both EEG and neuroimaging analyses, when strong hypothesis of the involved brain areas is possible. In fact, although the experimental task, seen as a whole, is brand new, the areas involved in the investigated interval have been accurately described in the past as producing a consistent picture that guided and supported our ROI selection. We adopted a conservative approach, selecting ROIs in areas on the outer surface of the brain where the spatial resolution of the EEG source analysis is maximal and avoiding the investigation of deep brain areas such as the proper FFA, orbitofrontal, para-hippocampal cortices and amygdala. These areas were reported in several neuroimaging studies but their reconstruction through EEG presents several methodological issues. EEG source analysis accuracy is in fact highly corrupted by the huge anisotropy and inhomogeneity of the brain that blur the emerging signal when it is not modeled by a proper volume conductor model. Deep sources are of course more buried within the brain as the ideal lines separating the sources from the scalp electrodes cross much more tissues of different conductivities than superficial sources, making the blurring much higher. Concerning the temporal selection, we opted to analyze up to ∼300 ms because we were interested in assessing the automatic processing of face stimuli, aware of the fact that the later components would have been altered by subjects’ intentions or strategies to concentrate on target stimulus (the word) decoding. We decided to investigate the task effect as a between-subject factor as we were interested in maximizing the “unattendeness” of face emotional expressions in the glasses shape task as much as possible. We feared that if half of subjects, due to the counterbalance of the task order, performed the emotional task first and then the glasses task, facial emotion might have acquired some relevance even when the glasses task asked subjects to attend and respond to only the glasses’ shape. In addition, we would have obtained an incredibly long task, with unpredictable consequences over subjects’ attention and performance level, with the risk of introducing undesired biases into our results. The failure to locate the areas that actually discriminate and extract the emotional features of the faces surely represents a limit of the present exploration. A trend versus a higher activation of pSTS in emotional compared to neutral expressions was found only in the emotion task. However, it was not significant even before applying the Benjamin and Yekuteli correction. This might be due to the spatio-temporal resolution of the method here implemented or, more presumably, because emotional processing also involves deep brain areas, such as FFA s, orbitofrontal cortices and subcortical regions.

## CONCLUSION

In the present study, we employed a novel approach to explore the role of attention in emotional face processing by setting up an ecological environment that involved faces wearing glasses. Moreover, by overlapping in space both the to-be-attended and the to-be-unattended facial features, we avoided any potential confounding produced by attention shifts, so that any emerging differences could be attributed more confidently to the availability of the attentional resources required to deal with facial emotional expressions. In studies that report emotional processing that was not affected by attentional manipulations, the emotion-unattended condition did not usually require investing a great amount of attentional resources; thus, it was difficult to claim that the emotional processing of faces could take place without attention. Here, consistent with our previous behavioral study (Sassi et al., 2014), in which emotion-irrelevant task demands were progressively increased, we observed that when subjects were involved in an emotion-irrelevant discrimination task that might have depleted attentional resources, behavioral results did not show any evidence of affective priming. These results corroborate the studies that support that emotional processing requires some attentional resources (Pessoa et al., 2002, 2005; Eimer et al., 2003; Holmes et al., 2003; Okon-Singer et al., 2007; Silvert et al., 2007). Importantly, although the attentional resources were allocated to detect the characteristics of the glasses, the angry facial expression activated the temporo–parietal area of the VAN. This automatic activation presumably represents a pre-attentive bottom–up trigger, possibly evoked by a subcortical pathway centered on the amygdala, which, independently from the ventral stream areas, signals the presence of unattended and task-irrelevant but potentially threatening stimuli (Ohman and Mineka, 2001). These results are in line with more recent reports (Cabeza et al., 2012) that disentangle TPJ activation from a re-orienting process that involves the DAN and can, for example, explain why search performance of angry faces is more efficient when they are displayed among several distractors (the anger superiority effect, Hansen and Hansen, 1988).

From an evolutionary point of view, the presence of such an early pre-attentive response, which also appears when subjects are comfortably seated in a safe environment, may increase the potential for a faster and more accurate identification of aversive emotional expressions (in the absence of proper inhibitory top–down signals aimed to ignore them, as in the present study). This mechanism would represent a successful adaptive process because a fast and correct prediction of aversive intentions may help the observers to better adapt their behavior and thus provide a crucial survival advantage (Frank and Sabatinelli, 2012).

## Conflict of Interest Statement

The authors declare that the research was conducted in the absence of any commercial or financial relationships that could be construed as a potential conflict of interest.
